# Association between eosinophil count and metabolically healthy obesity: A cross-sectional study based on NHANES 2005 to 2018

**DOI:** 10.1097/MD.0000000000049273

**Published:** 2026-06-12

**Authors:** Xue-Qin Zhang, Yin-Yi Ding

**Affiliations:** a Department of Food Science and Biological Engineering, School of Food Science and Biotechnology, Zhejiang Gongshang University, Hangzhou, Zhejiang Province, China.

**Keywords:** eosinophils, metabolically healthy obesity, metabolically unhealthy obesity, NHANES

## Abstract

Eosinophils, a pivotal component of the immune system, are known to contribute to both inflammatory responses and metabolic regulation. Nonetheless, the epidemiological link between eosinophil count and metabolically healthy obesity remains inconclusive. This study aims to examine the potential association between eosinophil levels and the odds of metabolically unhealthy obesity (MUO). The present analysis utilized data from the National Health and Nutrition Examination Survey 2005 to 2018 cycles, enrolling 4594 eligible obese adults. Participants were categorized as metabolically healthy obesity or MUO according to their metabolic status. Eosinophil counts, stratified by quartiles, served as the exposure variable. Weighted multivariate logistic regression and restricted cubic spline models were employed to evaluate their association with MUO odds. Additional subgroup and sensitivity analyses were performed to assess the robustness of the results. Among all obese participants, 84.33% were classified as MUO. Compared to the lowest quartile, individuals in the highest eosinophil count quartile had significantly elevated odds of MUO (adjusted odds ratio = 1.603, 95% confidence interval = 1.420–2.568, *P* < .001). When treated as a continuous variable, each 1 × 10^3^ cells/μL increment in eosinophil count was associated with a 2.13-fold higher odds of MUO. The restricted cubic spline analysis revealed a nonlinear association, with an inflection point around 0.1 (10^3^ cells/μL). The above associations were consistent across various subgroup and sensitivity analyses. Increased eosinophil levels were significantly associated with higher MUO odds in a nonlinear dose–response pattern. Eosinophil count may represent a promising epidemiological indicator for distinguishing metabolic obesity phenotypes.

## 1. Introduction

In recent years, the growing prevalence of obesity has emerged as a critical global public health concern. Data from the World Health Organization indicate that in 2022, 43% of adults aged ≥18 were overweight and 16% were obese globally.^[1]^ Although obesity is closely linked to insulin resistance, metabolic syndrome (MetS), and cardiovascular disorders, approximately 10% to 35% of obese individuals remain metabolically healthy – a phenotype known as metabolically healthy obesity (MHO).^[2–4]^ However, accumulating evidence indicates that MHO may be a transient state preceding metabolically unhealthy obesity (MUO), which still carries an elevated long-term risk for diabetes and cardiovascular complications.^[5–7]^ Studies indicate that nearly half of individuals with MHO progress to MUO within a few years, underscoring that MHO may not constitute a truly benign condition.^[8,9]^

Obesity is widely recognized as a state characterized by a chronic, high inflammatory burden,^[10,11]^ and these inflammatory and immune disturbances are key mechanisms contributing to obesity-induced metabolic dysfunction.^[12–14]^ Concurrently, an increment in blood eosinophil count is closely associated with increased inflammatory burden in certain clinical conditions.^[15,16]^ Therefore, exploring the variation of eosinophil counts across different obesity phenotypes is theoretically well-founded. Under obese conditions, sustained inflammation within adipose tissue impairs insulin sensitivity and fosters metabolic dysfunction.^[17,18]^ Eosinophils, as essential immune cells, have recently been shown to participate in modulating adipose tissue metabolism and inflammation.^[19,20]^ Evidence from animal and in vitro studies suggests that eosinophils promote an anti-inflammatory milieu in adipose tissue through the secretion of interleukin-4 (IL-4) and interleukin-13 (IL-13), thereby enhancing insulin sensitivity and inducing adipose tissue beiging.^[21–23]^ Conversely, epidemiological studies indicate that higher levels of circulating eosinophils are linked to a greater risk of MetS, highlighting the potentially complex role of eosinophils in the pathophysiology of obesity-related metabolic dysfunction.^[24,25]^

Although the link between eosinophils and obesity has been previously studied, few studies have specifically explored their relationship with the MHO phenotype. Previous evidence suggests that immune cell profiles in obese populations may contribute to differentiating metabolic health subtypes.^[26–28]^ Determining whether eosinophil levels differ between MHO and MUO may help elucidate the immunological mechanisms contributing to the metabolic heterogeneity of obesity. Therefore, using nationally representative data from the US National Health and Nutrition Examination Survey (NHANES) 2005 to 2018 cycles, this study aimed to investigate the association between eosinophil count and the MHO phenotype, with the goal of evaluating the potential utility of eosinophils as a marker of metabolic health and providing evidence to support early identification and intervention strategies for obesity subtypes.

## 2. Materials and methods

### 2.1. Data and sample sources

This study utilized data from the US NHANES conducted between 2005 and 2018. NHANES, organized by the National Center for Health Statistics, is a nationally representative cross-sectional survey aimed at evaluating the health and nutrition status of the US noninstitutionalized population. The survey employs a stratified, multistage probability sampling strategy to ensure that the sample is representative at the national level. All study protocols were approved by the ethics review board of the US Centers for Disease Control and Prevention, and informed written consent was obtained from all participants. The NHANES datasets are publicly accessible at https://www.cdc.gov/nchs/nhanes/.

**Figure 1.**
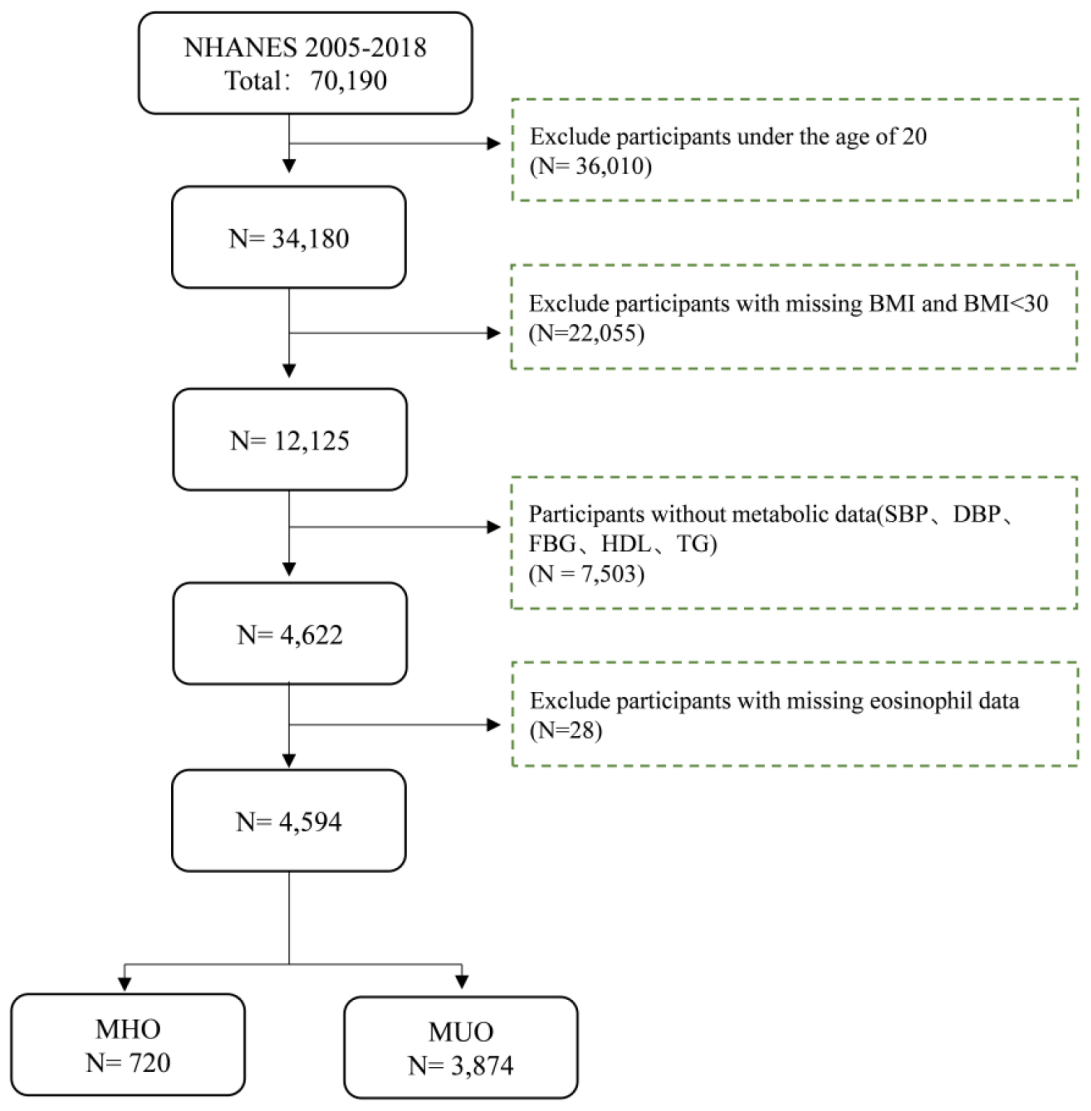
Flowchart of the participants selection process. BMI = body mass index, DBP = diastolic blood pressure, FBG = fasting blood glucose, HDL = high-density lipoprotein, MHO = metabolically healthy obesity, MUO = metabolically unhealthy obesity, NHANES = National Health and Nutrition Examination Survey, SBP = systolic blood pressure, TG = triglyceride.

The initial sample included 70,190 participants. Participants under 20 years old were first excluded, and those lacking data on key variables including body mass index (BMI), systolic blood pressure (SBP), diastolic blood pressure (DBP), fasting plasma glucose (FPG), high-density lipoprotein cholesterol (HDL-C), and eosinophil count were subsequently removed. Finally, a total of 4594 adults were included in the analysis, comprising 720 with MHO and 3874 with MUO, as defined below. The flowchart of sample selection is presented in Figure 1.

### 2.2. Exposure factors and outcome variables

The primary exposure variable was eosinophil count, derived from NHANES laboratory blood tests, expressed in 1000 cells per microliter (10^3^ cells/μL). Obesity was defined as having a BMI of 30.0 kg/m^2^ or greater.^[29]^ Metabolic health status was defined according to the harmonized criteria proposed by Lavie et al^[30]^ and Ortega et al^[31]^: participants with obesity who met none of the following 4 MetS components were classified as having MHO: elevated blood pressure (SBP ≥ 130 mm Hg, DBP ≥ 85 mm Hg, or current use of antihypertensive drugs); elevated FPG (≥100 mg/dL or current use of antidiabetic medication); low HDL-C levels (defined as <40 mg/dL for men and <50 mg/dL for women); elevated triglyceride levels (≥150 mg/dL). Participants with obesity who met 1 or more of these criteria were classified as MUO. In this study, metabolic phenotype was treated as the outcome variable, where MUO was coded as 1 and MHO as 0.

### 2.3. Covariates

Guided by prior literature and clinical relevance,^[32–35]^ this study adjusted for multiple potential confounders, including age, sex, race/ethnicity, education level, poverty-to-income ratio (PIR), low-density lipoprotein cholesterol, glycohemoglobin (HbA1c), oral glucose tolerance test (OGTT), total cholesterol, uric acid (UA), red blood cell (RBC), white blood cell (WBC), smoking status, alcohol consumption, hypertension, and diabetes. Race/ethnicity categories included Mexican American, non-Hispanic Asian, non-Hispanic Black, non-Hispanic White, other Hispanic, and other or multiracial individuals. Education was categorized into 3 levels: ≤high school, some college, and college graduate or above. PIR was divided into 3 strata: <1.30, 1.30 to 3.49, and ≥3.50. Individuals who reported smoking at least 100 cigarettes in their lifetime were classified as smokers. Alcohol consumption was defined as either drinking ≥12 alcoholic beverages in the past year or ever having consumed any form of alcohol. Hypertension was identified based on self-reported physician diagnosis or current use of antihypertensive medication. Diabetes was defined as the presence of any of the following: HbA1c ≥ 6.5%; fasting blood glucose ≥ 7.0 mmol/L; OGTT ≥ 11.1 mmol/L; physician-diagnosed diabetes; current treatment with antidiabetic medications or insulin. All covariates were obtained from NHANES and standardized prior to inclusion in the statistical models to minimize confounding bias.

### 2.4. Statistical methods

This study incorporated the complex survey design of NHANES in all analyses, using sampling weights to improve national representativeness and statistical reliability. The recommended sampling weights (WTMECPRP), pseudo-strata (SDMVSTRA), and pseudo-cluster variables (SDMVPSU) provided by National Center for Health Statistics were used to account for the multistage, stratified design of NHANES.

Participants were grouped according to their MHO/MUO phenotype and eosinophil count quartile distributions. Continuous variables were analyzed using weighted Student’s *t* tests or analysis of variance if normally distributed, and Mann–Whitney *U* tests or Kruskal–Wallis tests if non-normally distributed. Weighted chi-squared tests were applied for categorical variable comparisons. Continuous variables were presented as weighted mean ± standard deviation; categorical variables were described by unweighted counts and weighted proportions.

Stratified analyses using multivariable logistic regression were performed across subgroups defined by sex, race/ethnicity, education, and other covariates to assess effect modification. Three logistic regression models were developed to assess the association between eosinophil count and MUO, with multicollinearity assessed using variance inflation factors prior to model inclusion. Model 1 was unadjusted; Model 2 adjusted for age, sex, and race/ethnicity; Model 3 included additional adjustments for socioeconomic, biochemical, and clinical variables. Eosinophil counts were included both as continuous and categorical (quartile-based) variables, and results were expressed as odds ratios (ORs) with 95% confidence intervals (CIs).

Nonlinear associations between eosinophil count and MUO risk were evaluated using restricted cubic spline (RCS) modeling with data-driven knot placement and threshold effect estimation. Sensitivity analyses included alternative covariate specifications, exclusion of extreme values, and stepwise regression to assess the stability of the findings. A two-tailed *P*-value <.05 was considered statistically significant. All analyses were conducted using DecisionLinnc 1.0,^[36]^ with repeated validation to ensure reproducibility.

## 3. Results

### 3.1. Baseline characteristics of participants MHO and MUO

This study included 4594 eligible participants, whose average age was 48.63 years. Of the total population, 47.24% were male and 52.76% were female. Within the obese subgroup, 84.33% were identified as having MUO. Compared with individuals in the MHO group, those in the MUO group exhibited significantly elevated levels of BMI, SBP, DBP, FPG, HbA1c, OGTT, TG, UA, RBC, WBC, and eosinophil count (all *P* < .001), whereas HDL-C was significantly reduced (*P* < .001). Furthermore, significant differences were also observed between the 2 groups in terms of sex, race/ethnicity, educational attainment, PIR, BMI category, smoking status, hypertension, and diabetes prevalence (*P* < .05). Detailed results are presented in Table 1.

**Table 1:** Baseline characteristics of participants.

Characteristic	Overall (N = 4594)	MHO (N = 720)	MUO (N = 3874)	*P* value
Age (yr)	48.63 ± 15.64	42.16 ± 15.40	49.90 ± 15.38	<.001
BMI (kg/m^2^)	35.80 ± 5.51	34.62 ± 4.63	36.03 ± 5.64	<.001
SBP (mm Hg)	124.36 ± 16.07	118.73 ± 12.87	125.46 ± 16.40	<.001
DBP (mm Hg)	71.12 ± 12.25	68.82 ± 10.37	71.57 ± 12.53	<.001
FPG (mg/dL)	113.88 ± 38.00	92.60 ± 5.31	118.04 ± 40.18	<.001
HbA1c (%)	5.86 ± 1.13	5.32 ± 0.37	5.97 ± 1.20	<.001
OGTT (mmol/L)	7.11 ± 2.93	5.70 ± 1.55	7.44 ± 3.08	<.001
LDL-C (mg/dL)	116.09 ± 34.82	117.81 ± 32.36	115.74 ± 35.29	.109
HDL-C (mg/dL)	48.73 ± 13.28	57.86 ± 11.38	46.95 ± 12.89	<.001
TG (mg/dL)	152.16 ± 110.58	86.28 ± 28.80	165.04 ± 115.94	<.001
UA (mg/dL)	5.93 ± 1.41	5.45 ± 1.30	6.02 ± 1.41	<.001
TC (mg/dL)	193.89 ± 40.19	193.13 ± 35.12	194.04 ± 41.11	.817
RBC (million cells/µL)	4.76 ± 0.48	4.69 ± 0.49	4.78 ± 0.47	<.001
Eosinophils (1000 cells/µL)	0.22 ± 0.15	0.19 ± 0.14	0.22 ± 0.15	<.001
WBC (1000 cells/µL)	7.20 ± 2.11	6.56 ± 1.76	7.33 ± 2.15	<.001
Sex, n (%)				<.001
Male	2054 (47.24%)	257 (38.28%)	1797 (48.99%)	
Female	2540 (52.76%)	463 (61.72%)	2077 (51.01%)	
Race, n (%)				<.001
Mexican American	833 (9.99%)	108 (9.01%)	725 (10.18%)	
Other Hispanic	493 (5.76%)	69 (6.20%)	424 (5.67%)	
Non-Hispanic White	1932 (66.66%)	251 (60.13%)	1681 (67.94%)	
Non-Hispanic Black	1122 (13.14%)	254 (20.13%)	868 (11.78%)	
Other race	214 (4.44%)	38 (4.53%)	176 (4.42%)	
Education, n (%)				.003
Less than high school	1257 (18.89%)	129 (13.20%)	1128 (20.00%)	
High school or GED	1089 (24.00%)	164 (23.15%)	925 (24.17%)	
College or above	2248 (57.11%)	427 (63.64%)	1821 (55.83%)	
PIR, n (%)				.195
<1.30	1418 (21.17%)	184 (18.38%)	1234 (21.72%)	
1.30–3.49	2019 (42.78%)	339 (45.71%)	1680 (42.21%)	
≥3.50	1157 (36.04%)	197 (35.91%)	960 (36.07%)	
Diabetes, n (%)				<.001
No	3225 (75.48%)	703 (98.47%)	2522 (70.99%)	
Yes	1369 (24.52%)	17 (1.53%)	1352 (29.01%)	
Drink status, n (%)				.099
No	3875 (86.31%)	621 (88.74%)	3254 (85.83%)	
Yes	719 (13.69%)	99 (11.26%)	620 (14.17%)	
Smoke status, n (%)				<.001
No	2540 (54.66%)	489 (66.56%)	2051 (52.33%)	
Yes	2054 (45.34%)	231 (33.44%)	1823 (47.67%)	
Hypertension, n (%)				<.001
No	2394 (54.80%)	554 (80.37%)	1840 (49.80%)	
Yes	2200 (45.20%)	166 (19.63%)	2034 (50.20%)	

Categorical variables are presented as unweighted frequencies and weighted percentages, and group comparisons are performed using weighted chi-square tests. Continuous variables are presented as weighted means ± standard deviations, and group comparisons are performed using weighted ANOVA or weighted Kruskal–Wallis tests.

ANOVA = analysis of variance, BMI = body mass index, DBP = diastolic blood pressure, FPG = fasting plasma glucose, GED = General Educational Development, HbA1c = glycohemoglobin, HDL-C = high-density lipoprotein cholesterol, LDL-C = low-density lipoprotein cholesterol, MHO = metabolically healthy obesity, MUO = metabolically unhealthy obesity, OGTT = oral glucose tolerance test, PIR = poverty-to-income ratio, RBC = red blood cell, SBP = systolic blood pressure, TC = total cholesterol, TG = triglyceride, UA = uric acid, WBC = white blood cell.

### 3.2. Relationship between eosinophil count quartiles and baseline characteristics

To examine the dose–response association between eosinophil count and baseline characteristics, we stratified participants into 4 quartile groups (Q1–Q4) according to their eosinophil levels. Results indicated that individuals in the higher quartiles of eosinophil count had significantly elevated levels of BMI, SBP, FPG, HbA1c, OGTT, TG, UA, RBC, and WBC compared to those in the lower quartiles (*P* < .05). Conversely, HDL-C levels were markedly reduced (*P* < .001). In addition, significant differences were observed in sex, race/ethnicity, smoking status, diabetes, and hypertension across the quartiles (*P* < .001). Of particular note, the prevalence of MUO increased steadily across eosinophil quartiles – 77.60%, 85.04%, 85.23%, and 86.86% – indicating a potential link between elevated eosinophil count and MUO status (*P* < .001). Details are presented in Table 2.

**Table 2:** Baseline characteristics by eosinophil count quartiles.

Characteristic	Q1 (<0.1; N = 1148)	Q2 (0.1–0.2; N = 1148)	Q3 (0.2–0.3; N = 1148)	Q4 (>0.3; N = 1150)	*P* value
Age (yr)	47.21 ± 15.85	47.86 ± 15.02	50.19 ± 15.84	49.22 ± 15.65	<.001
BMI (kg/m^2^)	33.35 ± 3.02	38.11 ± 7.31	35.94 ± 4.76	35.95 ± 5.20	<.001
SBP (mm Hg)	123.18 ± 15.70	125.36 ± 17.28	124.66 ± 15.60	124.30 ± 15.69	.038
DBP (mm Hg)	71.06 ± 11.98	71.86 ± 12.27	70.79 ± 12.30	70.82 ± 12.41	.288
FPG (mg/dL)	110.02 ± 36.11	114.14 ± 37.97	115.55 ± 38.79	115.82 ± 38.82	<.001
HbA1c (%)	5.69 ± 1.04	5.87 ± 1.13	5.91 ± 1.10	5.97 ± 1.23	<.001
OGTT (mmol/L)	6.83 ± 2.74	7.28 ± 3.14	7.34 ± 2.91	7.01 ± 2.92	.015
LDL-C (mg/dL)	116.82 ± 34.87	117.14 ± 35.51	115.33 ± 34.82	115.13 ± 34.11	.541
HDL-C (mg/dL)	51.35 ± 13.87	49.52 ± 13.73	47.33 ± 12.15	46.77 ± 12.83	<.001
TG (mg/dL)	143.07 ± 126.41	148.74 ± 108.19	155.39 ± 98.48	161.23 ± 106.46	<.001
UA (mg/dL)	5.69 ± 1.39	5.91 ± 1.38	6.07 ± 1.42	6.05 ± 1.41	<.001
TC (mg/dL)	195.49 ± 40.78	194.96 ± 39.73	192.84 ± 40.74	192.33 ± 39.40	.289
RBC (million cells/µL)	4.72 ± 0.48	4.75 ± 0.47	4.77 ± 0.47	4.81 ± 0.49	<.001
WBC (1000 cells/µL)	6.52 ± 2.04	6.96 ± 2.08	7.35 ± 2.04	7.97 ± 1.99	<.001
Sex, n (%)					<.001
Male	470 (43.38%)	456 (42.30%)	523 (48.72%)	605 (54.23%)	
Female	678 (56.62%)	692 (57.70%)	625 (51.28%)	545 (45.77%)	
Race, n (%)					<.001
Mexican American	219 (10.42%)	202 (9.92%)	201 (9.32%)	211 (10.31%)	
Other Hispanic	114 (5.75%)	116 (5.91%)	118 (4.84%)	145 (6.55%)	
Non-Hispanic White	420 (63.39%)	441 (63.49%)	554 (71.88%)	517 (67.69%)	
Non-Hispanic Black	342 (16.40%)	339 (16.65%)	229 (10.27%)	212 (9.47%)	
Other race	53 (4.04%)	50 (4.02%)	46 (3.69%)	65 (5.98%)	
Education, n (%)					.568
Less than high school	303 (18.61%)	322 (20.01%)	321 (19.03%)	311 (17.99%)	
High school or GED	251 (21.80%)	269 (24.09%)	275 (25.43%)	294 (24.70%)	
College or above	594 (59.58%)	557 (55.90%)	552 (55.53%)	545 (57.32%)	
PIR, n (%)					.666
<1.30	320 (20.55%)	366 (22.10%)	345 (20.53%)	387 (21.58%)	
1.30–3.49	515 (42.17%)	504 (41.77%)	528 (45.53%)	472 (41.59%)	
≥3.50	313 (37.27%)	278 (36.13%)	275 (33.93%)	291 (36.84%)	
Diabetes, n (%)					<.001
No	882 (81.75%)	799 (75.12%)	769 (72.48%)	775 (72.54%)	
Yes	266 (18.25%)	349 (24.88%)	379 (27.52%)	375 (27.46%)	
Drink status, n (%)					.473
No	963 (86.90%)	961 (85.46%)	961 (85.27%)	990 (87.56%)	
Yes	185 (13.10%)	187 (14.54%)	187 (14.73%)	160 (12.44%)	
Smoke status, n (%)					<.001
No	703 (59.95%)	694 (62.23%)	591 (50.40%)	552 (46.56%)	
Yes	445 (40.05%)	454 (37.77%)	557 (49.60%)	598 (53.44%)	
Hypertension, n (%)					<.001
No	681 (60.98%)	586 (54.67%)	561 (53.50%)	566 (50.02%)	
Yes	467 (39.02%)	562 (45.33%)	587 (46.50%)	584 (49.98%)	
MUO, n (%)					<.001
No	251 (22.40%)	166 (14.96%)	157 (14.77%)	146 (13.14%)	
Yes	897 (77.60%)	982 (85.04%)	991 (85.23%)	1004 (86.86%)	

Categorical variables are presented as unweighted frequencies and weighted percentages, and group comparisons are performed using weighted chi-square tests. Continuous variables are presented as weighted means ± standard deviations, and group comparisons are performed using weighted ANOVA or weighted Kruskal–Wallis tests.

ANOVA = analysis of variance, BMI = body mass index, DBP = diastolic blood pressure, FPG = fasting plasma glucose, GED = General Educational Development, HbA1c = glycohemoglobin, HDL-C = high-density lipoprotein cholesterol, LDL-C = low-density lipoprotein cholesterol, MHO = metabolically healthy obesity, MUO = metabolically unhealthy obesity, OGTT = oral glucose tolerance test, PIR = poverty-to-income ratio, RBC = red blood cell, SBP = systolic blood pressure, TC = total cholesterol, TG = triglyceride, UA = uric acid, WBC = white blood cell.

### 3.3. Association between eosinophil count and MUO odds

To further investigate the association between eosinophil count and the odds of MUO, multivariable logistic regression models were employed (Table 3). In the unadjusted model (Model 1), eosinophil count, as a continuous variable, was significantly positively associated with MUO odds. Specifically, each 1 × 10^3^ cells/μL increment in eosinophil count was associated with a 2.13-fold increase in the odds of MUO (OR = 2.136, 95% CI = 1.672–2.949, *P* < .001). In the quartile-based analysis, individuals in the highest quartile (Q4) had 60.3% higher odds of MUO compared to those in the lowest quartile (Q1; OR = 1.603, 95% CI = 1.420–2.568, *P* < .001). After further adjusting for covariates, the association remained robust: Model 2 adjusted for age, sex, and race/ethnicity; Model 3 further accounted for potential confounders including education level, PIR, diabetes, and hypertension. The positive association between eosinophil count and MUO odds remained significant, suggesting that eosinophil count may be an independent factor associated with MUO.

**Table 3:** Association between eosinophil count and MUO risk.

Outcome	Model 1		Model 2		Model 3	
	OR (95% CI)	*P*	OR (95% CI)	*P*	OR (95% CI)	*P*
Eosinophils number	2.136 (1.672–2.949)	<.001	3.189 (1.376–4.957)	.007	2.383 (1.027–4.574)	.012
Eosinophils number (quartile)						
Q1	1 (reference)		1 (reference)		1 (reference)	
Q2	1.641 (1.223–2.204)	.001	1.653 (1.218–2.242)	.005	1.471 (1.087–1.986)	.012
Q3	1.665 (1.292–2.148)	<.001	1.477 (1.143–1.91)	.003	1.343 (1.033–1.74)	.028
Q4	1.603 (1.42–2.568)	<.001	1.706 (1.239–2.349)	.001	1.442 (1.057–1.966)	.021
*P* for trend		<.001		<.001		.038

Model 1 was without adjustment for covariates, Model 2 was adjusted for sex, age, and race, and Model 3 was adjusted for age, sex, race/ethnicity, education level, PIR, LDL-C, HbA1c, OGTT, TC, UA, RBC, WBC, smoking status, drinking status, hypertension, and diabetes.

CI = confidence interval, HbA1c = glycohemoglobin, LDL-C = low-density lipoprotein cholesterol, MUO = metabolically unhealthy obesity, OGTT = oral glucose tolerance test, OR = odds ratio, PIR = poverty-to-income ratio, RBC = red blood cell, TC = total cholesterol, UA = uric acid, WBC = white blood cell.

### 3.4. Subgroup analysis

**Figure 2.**
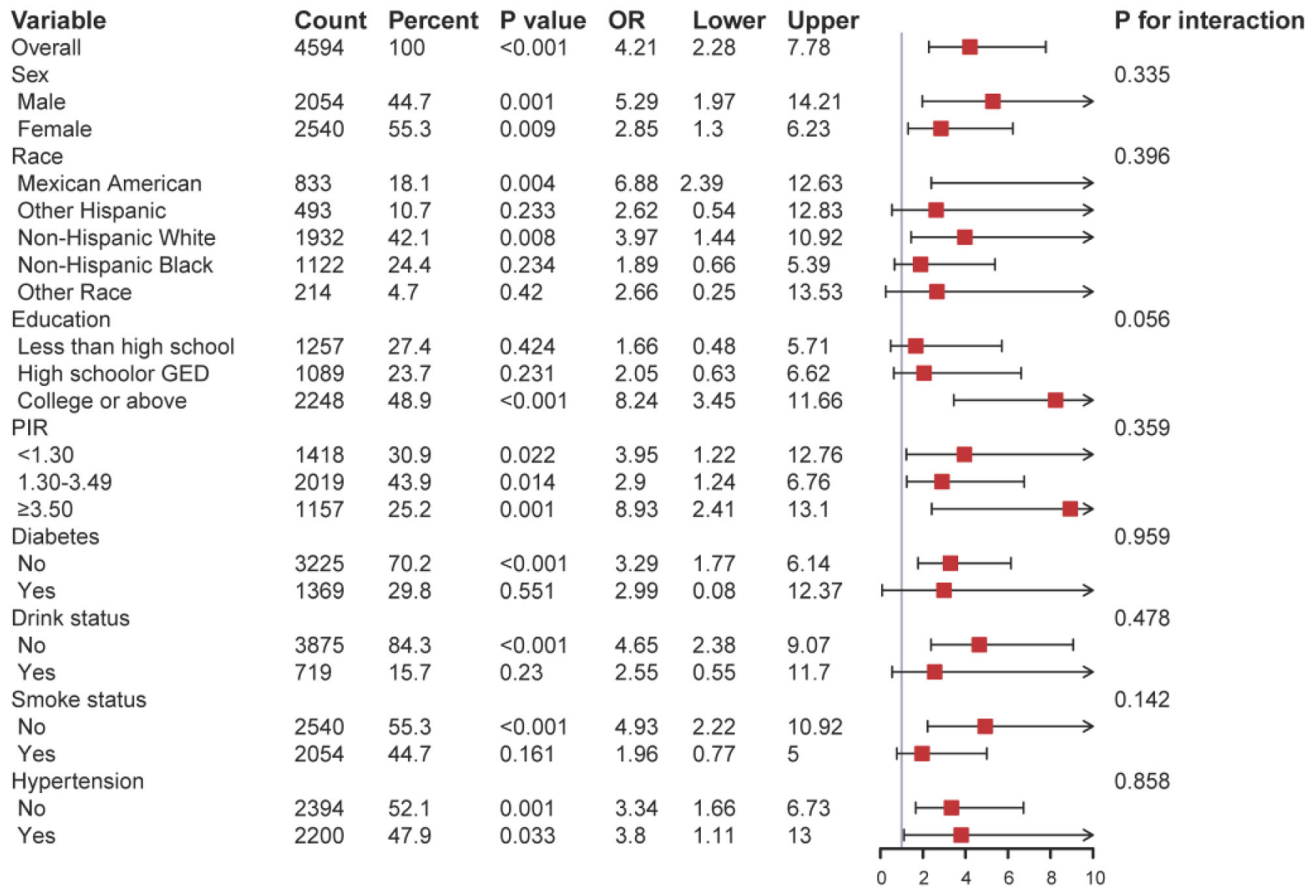
Subgroup analysis. OR = odds ratio, PIR = poverty-to-income ratio.

To further evaluate the association of eosinophil count with MUO odds across different populations, subgroup analyses were conducted based on sex, race/ethnicity, education level, PIR, smoking status, drinking status, diabetes, and hypertension. As shown in Figure 2, the positive association between eosinophil count and MUO odds remained consistent across all subgroups, and no significant interactions were observed (all *P* for interaction > .05). These results suggest that the positive association between eosinophil count and MUO is consistent across these studied demographic and clinical subgroups.

### 3.5. RCS analysis

**Figure 3.**
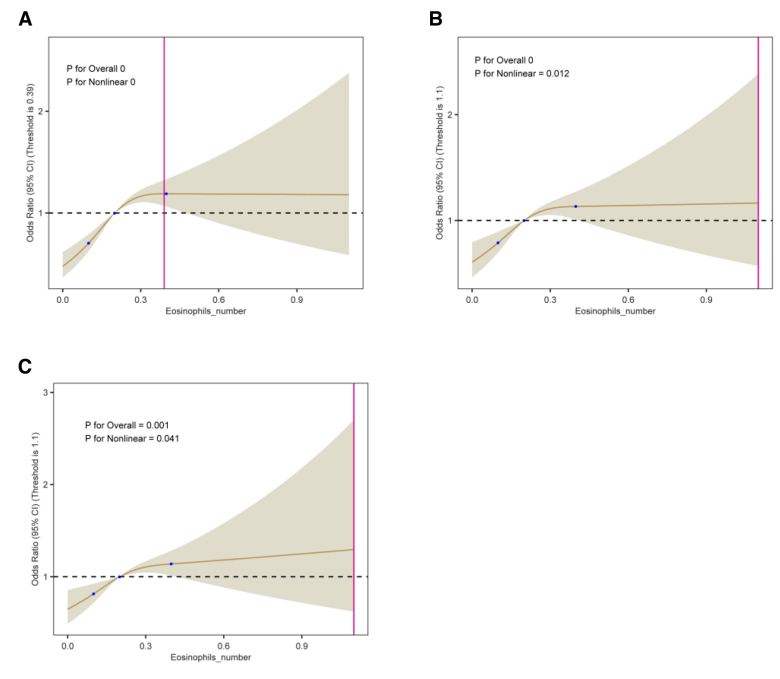
RCS analysis of the association between eosinophil count and the risk of MUO across different models. Each panel illustrates the fitted curves from the unadjusted model (A), the partially adjusted model (B), and the fully adjusted model (C). CI = confidence interval, MUO = metabolically unhealthy obesity, RCS = restricted cubic spline.

To further investigate the dose–response relationship and potential nonlinearity between eosinophil count and MUO odds, we fitted a RCS model. As shown in Figure 3, a significant nonlinear association between eosinophil count and MUO odds was observed in all models – unadjusted (Model 1), partially adjusted (Model 2), and fully adjusted (Model 3) – with consistent trends (*P* < .05).

The RCS analysis used knot locations set at 0.1, 0.2, and 0.4 across all 3 models. When the eosinophil count exceeded 0.2 (10^3^ cells/μL), the corresponding OR was >1, indicating a marked increase in MUO odds. Further threshold effect analysis revealed that the inflection point for eosinophil count consistently remained around 0.1 (10^3^ cells/μL) in all models. This suggests that the odds of MUO increase more prominently once the eosinophil count surpasses this inflection point.

### 3.6. Sensitivity and robustness analyses

To assess the robustness of the study findings, we conducted sensitivity analyses on both the logistic regression and RCS models. To minimize the potential influence of acute infections or underlying hematological disorders, we excluded participants with extreme blood cell counts (including eosinophils, WBC, and RBC) outside the 1st and 99th percentiles. Following these exclusions, the logistic regression results indicated that the positive association between eosinophil count and the odds of MUO remained statistically significant (*P* < .05). In the RCS analysis, the nonlinear associations also remained significant (*P* < .05) with consistent trends. These results validated the stability of the association between eosinophil count and the odds of MUO, strengthening the credibility of our conclusions.

## 4. Discussion

This study, based on nationally representative data from NHANES 2005 to 2018, investigated the association between eosinophil count and MHO. The results showed a significant positive association between eosinophil count and the odds of MUO, with the probability increasing progressively with higher eosinophil levels. Furthermore, RCS analysis confirmed a clear nonlinear dose–response relationship between eosinophil count and MUO odds, suggesting its potential as an associated epidemiological indicator for obesity metabolic phenotypes.

Currently, limited research has explored the relationship between eosinophils and metabolic obesity phenotypes, and existing findings remain inconsistent. Some animal and cellular studies have demonstrated that eosinophils secrete anti-inflammatory cytokines such as IL-4 and IL-13, promoting an anti-inflammatory adipose tissue environment and enhancing insulin sensitivity.^[20–23]^ However, epidemiological studies have suggested that elevated peripheral eosinophil counts are associated with increased metabolic burden or prevalence of MetS.^[37,38]^ Our findings support these epidemiological observations, suggesting a positive association between peripheral eosinophil counts and MUO in the general population.

The biological role of eosinophils in obesity-related metabolism has been hypothesized to exhibit dual characteristics. In local adipose tissue, eosinophils may promote the polarization of macrophages toward the M2 phenotype by releasing anti-inflammatory cytokines, thereby reducing insulin resistance^[39,40]^ and enhancing energy expenditure.^[41,42]^ Conversely, elevated eosinophil counts in peripheral blood may more likely reflect systemic inflammation or immune activation.^[43,44]^ Indeed, previous studies have linked elevated peripheral eosinophils with increased levels of other inflammatory markers (e.g., C-reactive protein, tumor necrosis factor-alpha) that indirectly contribute to metabolic disorders.^[45,46]^ However, such mechanistic interpretations must be approached with caution in the context of our findings. In the present work, we did not directly measure adipose tissue eosinophils, associated cytokines (e.g., IL-4, IL-13), C-reactive protein, tumor necrosis factor-alpha, or specific markers of insulin sensitivity. Therefore, definitively inferring a detrimental systemic role or detailed mechanisms based solely on our peripheral blood data exceeds the scope of this study. The precise relationship between eosinophils and metabolic health across different tissues requires further targeted molecular investigation.

The findings of this study have potential implications at the public health level by highlighting an epidemiological link between eosinophils and obesity subtyping. Nevertheless, a statistically significant association does not equate to clinical utility. Because our study did not evaluate predictive performance metrics (such as the area under the curve), perform comparisons with traditional metabolic markers, or conduct reclassification analyses, it is premature to suggest that eosinophil count should be incorporated into standard clinical workflows for metabolic risk assessment. Currently, it serves best as a supplementary epidemiological marker rather than a standalone clinical diagnostic tool.

Although this study is based on a large, nationally representative sample, several limitations remain. First, the cross-sectional design of NHANES prevents the establishment of a causal relationship or temporal sequence. Specifically, the possibility of reverse causality cannot be ruled out. Because MUO is characterized by severe insulin resistance, adipose tissue inflammation, and elevated cytokine levels, it is equally plausible that the metabolic dysfunction intrinsic to MUO drives systemic inflammation and reactive eosinophilia, rather than eosinophils driving MUO. Second, the prevalence of MUO in our study population was highly skewed (84.33%). This imbalance is a direct consequence of adopting a stringent “zero-abnormality” criterion for MHO. While clinically justifiable to capture true metabolic health, we did not test alternative definitions (e.g., allowing for 1 metabolic abnormality), which limits our ability to compare effect sizes across different diagnostic criteria. Finally, this study utilized peripheral eosinophil counts and lacked tissue-specific or detailed immunological data. Given the potential discrepancy between peripheral indicators and tissue microenvironments, future prospective studies involving the collection of human serum samples to directly quantify these systemic cytokines, alongside well-designed animal experiments, are essential to investigate the precise metabolic functions of eosinophils.

## 5. Conclusions

This study found that an elevated eosinophil count was significantly associated with higher odds of MUO, with a stable nonlinear dose–response relationship. The association was consistent across different populations, suggesting that eosinophil count may serve as an associated immunological indicator for distinguishing metabolic obesity phenotypes. Future longitudinal studies are warranted to confirm the causal relationship and to evaluate the predictive utility of eosinophil count in precision obesity management.

## Acknowledgments

We appreciate the NHANES databases for offering their platform and supplying valuable datasets.

## Author contributions

**Conceptualization:** Xue-Qin Zhang.

**Data curation:** Xue-Qin Zhang.

**Formal analysis:** Xue-Qin Zhang.

**Funding acquisition:** Xue-Qin Zhang.

**Investigation:** Xue-Qin Zhang.

**Methodology:** Xue-Qin Zhang.

**Supervision:** Yin-Yi Ding.

**Validation:** Yin-Yi Ding.

**Visualization:** Yin-Yi Ding.

**Writing – original draft:** Yin-Yi Ding.

**Writing – review & editing:** Yin-Yi Ding.
